# The Behavior and Postnatal Development in Infant and Juvenile Rats After Ultrasound-Induced Chronic Prenatal Stress

**DOI:** 10.3389/fphys.2021.659366

**Published:** 2021-04-15

**Authors:** Olga Abramova, Valeria Ushakova, Yana Zorkina, Eugene Zubkov, Zinaida Storozheva, Anna Morozova, Vladimir Chekhonin

**Affiliations:** ^1^Department of Basic and Applied Neurobiology, V.P. Serbsky National Medical Research Center for Psychiatry and Narcology, Moscow, Russia; ^2^Mental-health Clinic No. 1 Named After N.A. Alexeev of Moscow Healthcare Department, Moscow, Russia; ^3^Department of Biology, Lomonosov Moscow State University, Moscow, Russia; ^4^Department of Medical Nanobiotechnology, Pirogov Russian National Research Medical University, Moscow, Russia

**Keywords:** prenatal stress, ultrasound effect, rat offspring, anxiety, social behavior, postnatal rat development, maternal behavior, neonatal handling

## Abstract

Fetal development is susceptible to environmental factors. One such factor is exposure to stress during pregnancy. The present study aimed to investigate the effects of chronic prenatal stress (PS) on the development and behavior of rat offspring during infancy and juvenile ages. Existing approaches to modeling prenatal stress on animals do not correlate with the main type of stress in pregnant women, namely psychological stress. We used a new stress paradigm in the experiment, namely, stress induced by exposure to variable frequency ultrasound (US), which acted on pregnant Wistar rats on gestational days 1–21. This type of stress in rodents can be comparable to psychological stress in humans. We assessed physical development, reflex maturation, motor ability development, anxious behavior, response to social novelty, and social play behavior in male and female offspring. Additionally, we investigated maternal behavior and the effect of neonatal handling (NH) on behavior. Prenatal stress did not affect postnatal developmental characteristics in rat pups, but prenatally stressed rats had higher body weight in early and adult age than controls. Prenatal exposure to a stressor increased anxiety in the open-field test (OF), changed social preferences in the social novelty test (SN), and impaired social play behavior in males. Neonatal handling reduced anxiety and restored social behavior, but evoked hyperactive behavior in rat pups. Maternal behavior did not change. Our study demonstrated for the first time that exposure to variable frequency ultrasound during pregnancy influences offspring development and impairs behavior, correlating with the effects of other types of stress during pregnancy in rodents. This supports the idea of using this exposure to model prenatal stress.

## Introduction

Humans are exposed to many different adverse factors in modern society, such as unfavorable life events or poor environmental conditions, and which can induce a stress response in the organism. Pregnant women experience such negative influences, among others, which affect their offspring. Prenatal stress (PS) is the exposure of an expectant mother during pregnancy to stressors that affect the fetus indirectly through maternal stress (Fatima et al., 2017). This problem is very common. Estimates of the percentage of women who experience stress during pregnancy vary widely. For example, 8–12% of pregnant women meet the criteria for mental disorders during pregnancy in studies using clinical diagnostic tools. About 30% of pregnant women experience stress in everyday life according to other studies (Van den Bergh et al., 2017). Such exposure has an impact on offspring health, so research on the effects of PS on offspring is an extremely urgent task.

The brain is particularly sensitive to environmental influences during the prenatal period (Lautarescu et al., 2020). Maternal stress has a devastating effect on fetal brain development, leading to adverse cognitive, behavioral, and psychosocial effects in infancy and adulthood (Faa et al., 2016). PS is associated with the development of mental disorders, such as schizophrenia, depression, attention deficit hyperactivity disorder, and autism (Abe et al., 2007; Weinstock, 2016; Lautarescu et al., 2020). PS negatively affects the mental health of offspring, including at an early age. The first manifestations of many psychiatric disorders often begin in childhood or adolescence (Lewis et al., 2014). Up to 50% of all adult psychiatric disorders begin during adolescence (Belfer, 2008). Epidemiological studies have demonstrated that the rate of psychiatric disorders in children and adolescents is consistently between 13 and 20% (Lewis et al., 2014; von Klitzing et al., 2015).

According to animal studies, PS has long-lasting effects on offspring nervous system development in childhood and adulthood (Weinstock, 2016; Lautarescu et al., 2020). The practical implementation of PS in animals includes exposure of pregnant animals to specific external stimuli at specific stages of pregnancy or during the birth of the offspring. Such stimuli include physiological and psychological stress, immune activation agents, nutrient deficiencies, and complications during childbirth (Meyer and Feldon, 2010). In human terms, pregnant women are exposed to psychological stress more often than physical stress. Although pregnant women may be physically injured or suffer an infection, they are more likely to experience psychological stress, such as worrying about their babies, financial problems, difficulties at work, and so on. Consequently, animal models in which the effects caused by psychological stress, but not physical stress, can be evaluated should be used to study the etiology of PS-induced psycho-neurological disorders in humans (Abe et al., 2007). One commonly used procedure for inducing psychological stress in pregnant female rodents is restraint stress. In this experimental approach, pregnant animals are placed in restrainers one or more times a day for a certain period of gestation. Another frequently used approach is chronic unpredictable stress. The main feature of such stress protocols is a daily change in the form of stress over a long period of time. Such types of stress as restraining stress, deprivation of water or food, swimming in cold water, and exposure to loud noise are used (Meyer and Feldon, 2010). These approaches are widely used in studies, but it does not focus on the main stressor that affects a person in modern society, namely the impact of information stress. The use of ultrasound (US) seems promising for this task. Rodents emit US signals with a frequency of 22–25 kHz in the presence of danger, after being struck in a fight, in pain. Rat pups emit 40 kHz signals when isolated from dams (Takahashi et al., 2010). Unavoidable exposure of adult rats to US variable frequencies (22–40 kHz) induces a state of stress in them, and when chronically exposed for 3 weeks, the animals develop a depressive-like state, as previously demonstrated (Morozova et al., 2013a,b, 2016; Zorkina et al., 2019). Therefore, we hypothesized that chronic exposure of pregnant females to variable US throughout the gestation period (21 days) would cause a stress response in them, which in turn would lead to developmental disorders in the offspring, including abnormalities in nervous system development and behavior.

We aimed this study to investigate the effect of PS induced by chronic exposure to variable frequency US on the physical development and behavior of juvenile rat offspring. We examined physical development, reflex development, anxious behavior, responses to social novelty, and social play behavior in rat pups. We also broached the topic of the effect of neonatal handling (NH) on rat behavior. The effect of PS on the offspring may be due not only to physiological changes in the mother’s organism under stress, but also to altered maternal behavior and the relationship between the dams and pups. Therefore, in this study, we also investigated the effect of PS on maternal behavior traits.

## Materials and Methods

### Animals

We used Wistar rats from the Nursery for Laboratory Animals (Pushchino, RAS, Moscow region) in the experiment. All animals were kept at a constant temperature (23°C) with controlled direct lighting (12/12 h) and free access to water and food. Housing conditions and all experimental procedures were set up and maintained in accordance with Directive 2010/63/EU of 22 September 2010 and approved by the local ethical committee of V.P. Serbsky National Medical Research Center for Psychiatry and Narcology.

### Design of the Experiment

The female rats (*n* = 29) were divided into two groups. The rats of the experimental group (*n* = 15) were kept in individual cages (53 cm × 35 cm × 19 cm) after fertilization and throughout the pregnancy (21 days) and were exposed to US produced by an US generator. The US exposure was performed for 24 h each day during 3 weeks and consists of periods between the following range: low frequencies (20–25 kHz), middle range frequencies (25 < × < 40 kHz), and frequencies of high range (40–45 kHz). The ultrasound frequencies changed every 10 min. Low and middle frequency ultrasound constituted 35% of emission each, high frequencies constituted 30% of emission time. The loudness of the sound was fluctuating at the range ±10% of the averaged value, i.e., 50 ± 5 dB. The ultrasonic device was suspended from the ceiling, and the loudspeaker was oriented downwards, where there were cages with rats at a distance of 1.5 m. Technical specifications of US generator: 220 V with adapter (0.3 W). The position of the cages was changed every 3 days. The rats exposed to US were kept in the separate room in equal conditions with control rats. After pregnancy, the dams with offspring were kept in individual cages under normal conditions without US. Control females (*n* = 14) were kept under normal conditions in individual cages without US impact throughout pregnancy. After pregnancy, control dams with offspring were kept in individual cages (53 cm × 35 cm × 19 cm) under normal conditions. As a result, one stressful female did not become pregnant or give birth, one control female died in childbirth, and another control female gave birth to dead pups. Two offspring groups were obtained from 26 females for the experiment: experimental group [PS offspring; 49 males (PS males) and 58 females (PS females)] born by stressed mothers and control group [C offspring; 60 males (С males) and 59 females (C females)] born by control mothers. The pups were weaned at 22 days of age and housed in groups (≤8 rats per cage).

**Figure 1:**
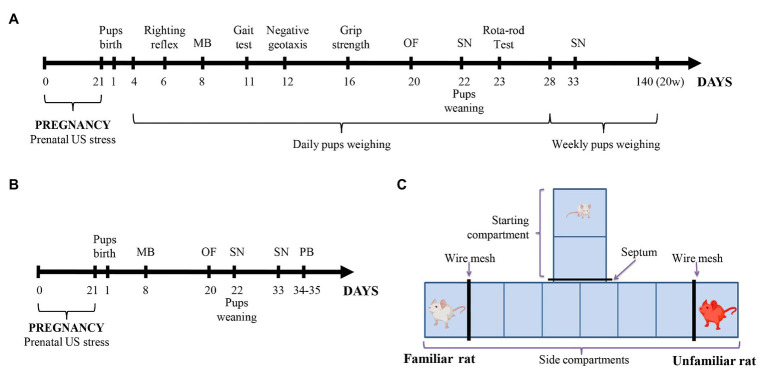
**(A,B)** Experimental design. Pregnant Wistar rats were subjected to a variable frequency ultrasound (US) stress from gestational day 1 to 21. Control pregnant rats were kept under normal conditions without US impact. Physical development, reflex maturation, and behavior were assessed in one part of the offspring **(A)**. In the other part of the offspring **(B)** only behavior was assessed. **(C)** The T-maze scheme for the social novelty test. MB, maternal behavior test; OF, open-field test; PB, social play behavior test; SN, social novelty test; and US, ultrasound; and w, weeks.

The scheme of the experiment is shown in Figures 1A,B. The offspring of six control dams and five stressful dams were only testing in behavioral tests from 20 postnatal days (PNDs; Figure 1B). We used tests such as open field (PND 20), social novelty test (SN; PND 22 and 33), and play behavior test (PND 34–35). Physical developmental parameters (PND 1–20), body weight, parameters of neurological reflexes, and motor coordination (PND 6–23) were recorded for the remaining offspring (the offspring of six control dams and nine stressful dams; Figure 1A). We hypothesized that this exposure affects behavior, as many studies support the effects of NH on behavior (Papaioannou et al., 2002; Raineki et al., 2014). We performed an open-field test (OF; PND 20) and a social novelty test (PND 22 and 33) with these rat offspring to examine the effects of NH on anxiety and social behavior in them. The handled offspring was designated as “hPS” and “hC” for PS and control offspring (C), respectively.

All tests were conducted from 10.00 to 13.00. Animal behavior in all tests was recorded with a digital video camera and analyzed using RealTimer software (OpenScience).

### Maternal Behavior Test

The maternal behavior test (MB) was performed with dams on PND 8 according to protocol (Dobryakova et al., 2011). The round open field arena (diameter 100 cm, wall height 12 cm) was used for the test. Squares with sides 25 cm × 25 cm marked the bottom of the arena. A Petri dish was placed in the center of the arena. Preliminarily, the females adapted in the experiment room within 30 min. The test was conducted in three stages. In the first stage, the females were placed on the edge of the arena in red light. Horizontal activity (total squares crossed), vertical activity (rearing frequency), and grooming [frequency, total time (s), and mean duration (s)] were recorded for 2 min. The female was resting in a home cage with the pups for 1 min after the trial. In the second stage, three female’s pups were placed in a Petri dish in the center of the arena. The female was placed on the edge of the arena in a red light, and maternal behavior was recorded for 2 min: latency (s) of the first approach to the dish with pups, number of approaches to the dish (s), total time around the dish (s), number of pups transferred to another place, and latent transfer time of each pup (s). The female was resting in a home cage with the pups for 1 min after the trial. In the third stage, three pups were also placed in the center of the arena. The female was placed on the edge of the arena in bright white light (140 lux). The same parameters of maternal behavior were recorded for 2 min.

### Physical Developmental Parameters

The pups were examined daily until the weanling day (PND 22). The number of newborn rat pups was recorded on PND 1. Maturation of physical characteristics for the whole litter was inspected by recording the PND of fur appearance, pinna detachment (the opening of ear channel), eye-opening (opening of both eyes lids), and lower incisors eruption. Weight (g) of offspring was recorded every day from PND 4 until PND 28. Weight of offspring was also recorded every week until 20 weeks of age after PND 28.

### Development of Neurological Reflexes and Motor Coordination

A number of tests were performed on certain PNDs to study the maturation of sensory and motor reflexes in rat pups before weaning.

#### Righting Reflex (PND 6)

This test is believed to be a reflection of muscle strength and subcortical maturation (Fan et al., 2008). The pup was placed on his back on a flat surface and released quickly. The time (s) required to turn over on all 4 ft and touch the surface was measured. The test was limited to 30 s.

#### Gait Test (PND 11)

For the experiment, a surface with a circle marked on it and a diameter of 13 cm was used. The pup was placed in the center of the circle and the time (s) required for the pup to move out of the circle with all 4 ft was recorded. The test was limited to 120 s.

#### Negative Geotaxis (PND 12)

This test is believed to test reflex development, motor skills and vestibular labyrinth, and cerebellar integration (Kiss et al., 2005; Fan et al., 2008). The pup was placed on an inclined surface (45° inclined surface angle, length 30 cm) with his head down. The time (s) spent for a turn of 180° upward was recorded. The test was limited to 120 s.

#### Grip Strength (PND 16)

This maneuver tests neuromuscular and locomotor development (Fan et al., 2008). The pup was placed on a vertical wooden rod (rod length 15 cm, diameter 1 cm). The total time (s) during which the pup was able to hold onto the rod was recorded.

#### Rota-Rod Test (PND 23)

The pup was previously trained to hold on a 4 cm diameter rotating cylinder at 7 rpm for 1 min. After 30 min of training, the holding time (s) on the rotating cylinder at 20 rpm was recorded. The test was limited to 180 s.

### Open-Field Test

The open-field test was conducted on a square arena (24 cm × 24 cm × 23 cm) on PND 20. The bottom of the arena was divided into nine squares (8 cm × 8 cm). The arena was illuminated with direct light of 140 lux. The arena was cleaned after each animal. The animal was placed in a certain corner of the arena at the beginning of testing and its behavior is recorded over 5 min (Zorkina et al., 2019). The following parameters were recorded: arena center crossing frequency, horizontal activity (total squares crossed), vertical activity (rearing frequency), the total time of freezing (s), the grooming total time (s), grooming frequency, and mean grooming duration (s).

### Social Novelty Tests (Dam/Unfamiliar Female and Sibling/Non-sibling)

The social novelty test was performed to study the social preference between familiar and unfamiliar rats in T-maze. The test was performed with a dam and an unfamiliar non-lactating female on PND 22, and with a sibling rat and non-sibling rat on PND 33, in red light (4 lux), according to the protocol described in Dobrovolsky et al. (2019). The T-maze (Figure 1C) had 35 cm high opaque walls, 10 cm × 26 cm starting compartment, and 10 cm × 50 cm two side compartments. The bottom of the maze was marked with equal 10 cm × 13 cm squares (two squares in the starting compartment and six squares in the side compartments) for horizontal activity evaluation. Each dead-end of the side compartments was separated by a wire mesh to form a 10 cm × 15 cm angle for a familiar or unfamiliar rats. Before starting the test, familiar and unfamiliar rats were placed behind the wire mesh in the side compartments. The starting compartment was closed by a septum. The experimental rat was placed in the closed starting compartment for 60 s. The septum was removed and behavior is fixed for 5 min. The following parameters were recorded: horizontal activity (total squares crossed), vertical activity (rearing frequency), the latency of exit from the starting compartment (LE; s), the latency of social contact (s) and the total time of social contact (s), and also the latency of contact (s) with familiar or unfamiliar rats. The standing time (s) in the central compartment (Tc), in the side compartment with a familiar rat (T1), and in the side compartment with an unfamiliar rat (T2) were recorded. The percentage of standing time in each compartment was estimated from the total time remaining after the exit from the starting compartment. Therefore, the standing time in the central compartment was calculated by a formula:

Tc=Totaltimespentinthecentralcompartment−LE

The percentage of standing time in i-compartment (P) was calculated by a formula:

$$Pi=\frac{Ti}{Tc+T2+T1}*100$$

### Social Play Behavior Test

Playing behavior was assessed according to the protocol described in (Cutuli et al., 2019) with adolescent rat pups (PND 34–35). On the first day of the experiment, the pups were habituated to an experimental room by having been exposed for 4 h. On the second day of the experiment, the pups were marked on their backs and placed in individual cells for 3.5 h to stimulate playful behavior. The pups were then placed in pairs in a neutral cage (35 cm × 56 cm × 18 cm) with fresh bedding. The tested pairs encompassed same-sex partners belonging to the same group of offspring. The partners were not litter‐ or cage mates. The test was performed for 15 min in a red light. Duration (s), frequency, and latency (s) of the social exploration (sniffing or grooming) and play behavior were scored. The elements of play behavior (pouncing, pinning, boxing, and chasing) were scored in a similar manner.

### Statistics

The RStudio was used for statistical analysis. The normal distribution was verified by the Shapiro-Wilk normality test. In case of normal distribution, values of *p* were calculated by using Student’s *t* test for pair wise comparisons, and ANOVA followed by Tukey’s test for multiple comparisons, the results were presented as Mean ± SEM; otherwise, Kruskal-Wallis rank sum test and Mann–Whitney U test were used, and the results were presented as Median (Q1; Q3). A GLM ANOVA was performed using body weight as a covariate. A value of*p* < 0.05 was considered statistically significant.

## Results

### Maternal Behavior

Maternal behavior test revealed no differences in maternal behavior parameters between control and stressed dams in the second and third stages of the test. However, in the first stage, stressed dams had less vertical activity compared to control dams (*F* = 5.38, *p* = 0.03). Stressed dams had an average of 7.2 ± 0.8 rears during first stage of the test; control females had an average of 10.5 ± 1.2 rears. Horizontal activity and grooming did not differ at the first stage.

### Physical Developmental

We found that dams stressed during pregnancy gave birth to fewer pups than control dams (*U* = 117, *p* = 0.03). C and PS offspring did not differ significantly in the days of fur appearance, lower incisors eruption, pinna detachment, and eye-opening (Table 1).

**Table 1:** Physical developmental parameters of the offspring.

Parameters	C	PS	*N*	*p* value
Number of newborn rat pups at 1 PND	11.0 ± 0.5	7.5 ± 0.9	26	**0.03**
Fur appearance for the whole litter, PND	7.0 ± 0.5	7.0 ± 0.2	15	0.86
Lower incisors eruption for the whole litter, PND	11.1 ± 0.3	12.0 ± 0.4	15	0.44
Pinna detachment for the whole litter, PND	13.5 ± 0.3	14.0 ± 0.1	15	0.22
Eye-opening for the whole litter, PND	17.5 ± 0.7	17.0 ± 0.2	15	0.31

PND, postnatal day; PS, prenatal stress offspring; and C, control offspring. Statistically significant values (p < 0.05) are highlighted in bold.

Analysis of offspring body weight gain revealed large differences between control and experimental offspring. PS males had significantly higher body weight compared to C males starting at PND 6 (Table 2). PS females also had a higher body weight than C females until 9 weeks of age, but these differences were less pronounced. After 9 weeks of age, PS and C females did not differ in weight (Table 2).

**Table 2:** Offspring body weight.

Time point	Males		*p* value	Females		*p* value
	**C**	PS		C	PS	
PND 5	9.8 ± 0.2	10.5 ± 0.4	0.21	9.2 ± 0.2	9.6 ± 0.3	0.45
PND 6	10.7 ± 0.2	12.1 ± 0.3	**0.008**	10.6 ± 0.3	11.2 ± 0.3	0.40
PND 7	11.4 ± 0.3	13.4 ± 0.3	**<0.001**	11.0 ± 0.4	12.4 ± 0.3	**0.01**
PND 9	12.5 ± 0.4	14.8 ± 0.4	**0.003**	12.3 ± 0.6	14.5 ± 0.3	**0.002**
PND 11	16.2 ± 0.3	20.0 ± 0.4	**<0.001**	16.4 ± 0.5	18.1 ± 1.1	**<0.001**
PND 14	20.2 ± 0.3	22.2 ± 1.2	**0.04**	20,5 ± 0.5	21.8 ± 0.9	0.27
PND 16	19.0 ± 0.8	24.4 ± 1.0	**<0.001**	17.5 ± 0.9	24.1 ± 0.5	**<0.001**
PND 19	23.6 ± 0.4	29.1 ± 0.8	**<0.001**	23.2 ± 0.5	27.7 ± 0.6	**<0.001**
PND 22	27.9 ± 1.0	37.1 ± 0.8	**<0.001**	27.9 ± 1.3	34.7 ± 0.8	**<0.001**
PND 25	33.5 ± 2.0	42.7 ± 1.6	**0.03**	36.0 ± 2.3	38.7 ± 2.0	0.46
PND 28	39.6 ± 1.2	50.3 ± 2.1	**<0.001**	40.8 ± 1.7	48.2 ± 1.7	**0.01**
w 5	59 ± 2.0	78 ± 2.7	**<0.001**	63 ± 3.0	77 ± 2.1	**<0.001**
w 6	84 ± 3.1	99 ± 3.4	**0.03**	94 ± 4.2	99 ± 3.1	0.74
w 7	103 ± 3.7	131 ± 4.4	**<0.001**	119 ± 5.0	128 ± 4.0	0.19
w 8	134 ± 4.6	165 ± 5.9	**<0.001**	135 ± 6.2	155 ± 4.2	**0.04**
w 9	154 ± 6.0	198 ± 7.2	**<0.001**	155 ± 5.2	172 ± 4.0	**0.03**
w 10	189 ± 5.5	237 ± 7.2	**<0.001**	172 ± 4.8	189 ± 4.2	0.13
w 11	208 ± 5.9	261 ± 7.3	**<0.001**	188 ± 5.0	193 ± 4.7	0.91
w 12	225 ± 6.3	282 ± 6.6	**<0.001**	192 ± 5.0	203 ± 4.0	0.44
w 13	245 ± 7.0	307 ± 6.8	**<0.001**	202 ± 5.0	215 ± 4.0	0.42
w 18	319 ± 6.0	368 ± 7.5	**<0.001**	233 ± 4.6	243 ± 3.6	0.48
w 20	347 ± 6.2	385 ± 10.8	**<0.001**	250 ± 6.1	265 ± 5.3	0.10

PND, postnatal day; PS, prenatal stress offspring; C, control offspring; and w, week. Statistically significant values (p < 0.05) are highlighted in bold.

### Development of Neurological Reflexes and Motor Coordination

We found no difference between the C and PS offspring in the parameters of maturation of sensory-motor reflexes and motor behavior (Table 3).

**Table 3:** Maturation parameters of neurological reflexes and motor coordination.

Parameters	Males		Females		p-value
	C	PS	C	PS	
The time required to turn over (righting reflex), s	1.8 ± 0.2	1.6 ± 0.2	1.7 ± 0.1	1.8 ± 1.1	0.96
The time required for move out of the circle (gait test), s	120.0 ± 7.7	120.0 ± 7.0	105.0 ± 7.6	104.0 ± 7.3	0.80
The time spent for a turn (negative geotaxis), s	8.8 ± 1.7	9.1 ± 1.4	10.1 ± 1.2	7.0 ± 0.9	0.18
The total time for hold (grip strength), s	18.0 ± 3.7	22.0 ± 2.4	17.0 ± 5.4	17.0 ± 2.7	0.51
The holding time on the rotating cylinder (rota-rod test), s	62.0 ± 11.2	41.0 ± 13.2	90.0 ± 13.5	71.0 ± 11.2	0.18

PS, prenatal stress offspring; C, control offspring.

### Open-Field Test

**Figure 2:**
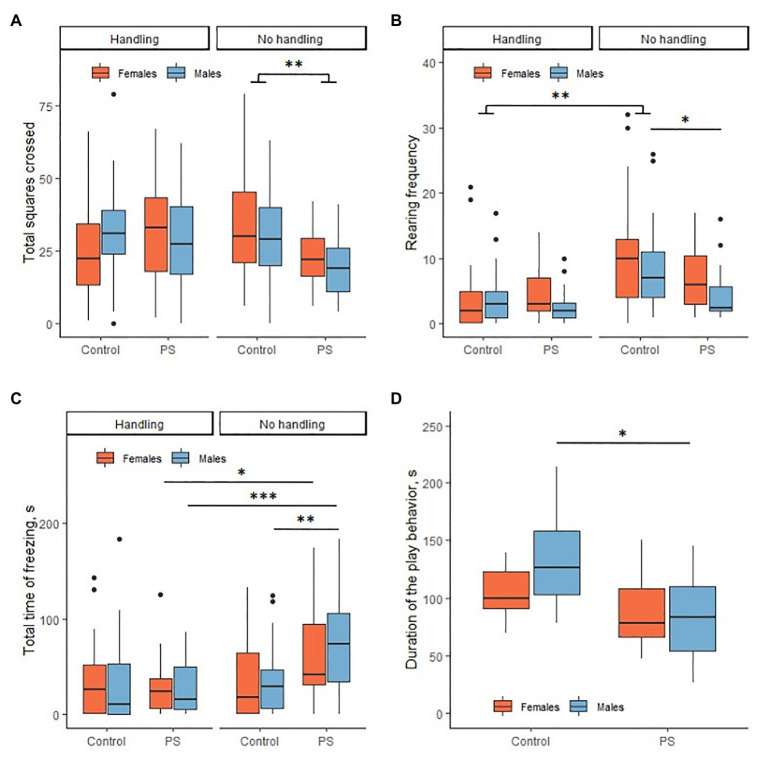
Effects of prenatal stress on locomotor activity, anxiety, and social play behavior. **(A)** Prenatal stress reduced horizontal activity in the offspring in the open-field test. **(B)** Prenatal stress reduced vertical activity in males in the open-field test. Neonatal handling (NH) decreased vertical activity in control rats. **(C)** Prenatal stress increased the total time of freezing in males in the open-field test. NH decreased the total freezing time in PS offspring to the control level. **(D)** Prenatal stress reduced the duration of the play behavior in males. ^*^p < 0.05; ^**^p < 0.01; ^***^p < 0.001; Control, control rat offspring; PS, rat offspring with prenatal stress.

Prenatal stress had an effect on the results of the open-field test in the offspring. Statistically significant differences were found in the horizontal and vertical activity and total time of freezing. PS offspring generally had less horizontal activity compared to C offspring (*F* = 7.09, *p* = 0.010). PS offspring had 23.5 ± 2.2 squares crossed on average, control offspring 31.3 ± 2.4 squares crossed (Figure 2A). There were no sex differences in the horizontal activity. We noted a decrease in vertical activity in PS males as compared to С males (*U* = 121, *p* = 0.01). PS males had an average of 2.5 (2; 5.7) rears; C males had 7 (4; 11) rears (Figure 2B). In addition, PS males exhibited higher total time of freezing than С males (*U* = 338.5, *p* = 0.005). The total time of freezing in PS male averaged 79.9 ± 13.1 s, in C males 29.5 (6.8; 47.4) s (Figure 2C). Females demonstrated no differences in the vertical activity and total time of freezing. Additionally, the offspring had no statistically significant differences in the center crossing frequency and grooming.

### Social Novelty Test

#### Dams/Unfamiliar Non-lactating Females (PND 22)

**Figure 3:**
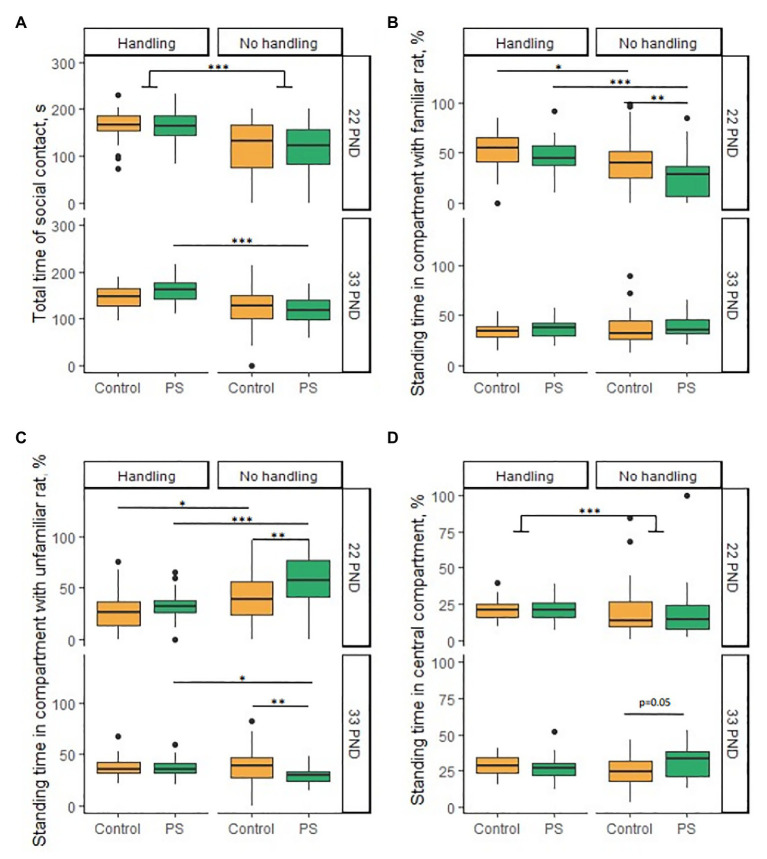
Effects of prenatal stress and neonatal handling on social behavior in the social novelty test on PND 22 and 33. **(A)** Neonatal handling increased total time of social contact in almost all groups on PND 22 and in PS offspring on PND 33. **(B)** PS offspring spent less time (%) in the compartment with the dams compared to the control on 22 PND. Neonatal handling increased the standing time (%) in the compartment with dams in the PS and control offspring on PND 22. **(C)** PS offspring spent more time (%) in a compartment with an unfamiliar female rat on PND 22 and less time in a compartment with non-siblings on PND 33 compared to control. Neonatal handling reduced the time (%) in a compartment with an unfamiliar female rat in PS and control offspring on PND 22. Neonatal handling increased the standing time (%) in the compartment with non-siblings in PS offspring to a control level on PND 33. **(D)** PS offspring spent more time (%) in the central compartment (*p* = 0.05) than control offspring on PND 33. Neonatal handling increased standing time (%) in the central compartment in offspring on PND 22. ^*^p < 0.05; ^**^p < 0.01; ^***^p < 0.001; Control, control rat offspring; PS, rat offspring with prenatal stress.

C and PS offspring did not differ from each other in the horizontal and vertical activity, LE, total time of social contact, and the latency of contact with familiar or unfamiliar rats. We also found no sex differences in this test. However, we found a significant difference between the C and PS offspring in the percentage of standing time in the compartment with the dams, even though there were no differences in the total time of social contact. In general, PS offspring spent a lower percentage of time in the dam compartment compared to C offspring (*F* = 8.11, *p* < 0.001; PS offspring 25.7 ± 3.8%, С offspring 39.6 ± 4.3%; Figure 3B). Tukey’s pairwise comparisons revealed no intergroup differences separately for males (*p* = 0.12) and females (*p* = 0.38). Also, PS offspring spent a higher percentage of time in the compartment with unfamiliar female rats compared to C offspring (*F* = 17.95, *p* < 0.001; PS offspring 56.1 ± 4.6%, C offspring 40.3 ± 4.2%; Figure 3C). However, Tukey’s pairwise comparisons also revealed no intergroup differences separately for males (*p* = 0.35) and females (*p* = 0.16). C and PS offspring did not differ significantly in the percentage of standing time in central compartment.

#### Sibling Rats/Non-sibling Rats (33PND)

C and PS offspring did not differ from each other in the horizontal and vertical activity, LE, total time of social contact, latency of contact with familiar or unfamiliar rats, and the percentage of standing time in compartment with siblings. We found no sex differences in this test either. Overall, PS offspring spent a lower percentage of time in the non-sibling compartment compared with C offspring (*F* = 5.44, *p* = 0.023; PS offspring 30.2 ± 1.6%, C offspring 39.0 ± 2.9%; Figure 3C). However, Tukey’s pairwise comparisons revealed no intergroup differences separately for males (*p* = 0.08) and females (*p* = 0.36). We observed overall group differences equal to the level of significance in the percentage of standing time in central compartment (*F* = 4.0, *p* = 0.05). PS offspring spent a higher percentage of time in central compartment (30.8 ± 2.0%) than C offspring (25.5 ± 1.7%; Figure 3D). However, Tukey’s pairwise comparisons revealed no intergroup differences separately for males (*p* = 0.23) and females (*p* = 0.81).

### Social Play Behavior Test

The social play behavior test (PB) was performed only with non-handled offspring. We found significant differences in the duration of the play behavior, PS offspring played less overall than C offspring (*F* = 9.6, *p* = 0.003). This difference was determined by the difference in male duration of play behavior (*p* = 0.01; Figure 2D). C males spent an average of 134.2 ± 13.5 s to play, PS males 84.8 ± 10.7 s. C and PS females had no differences (*p* = 0.70). C females spent an average of 104.7 ± 6.8 s to play, PS females 88.6±9.8 s. PS males had a reduced frequency of play behavior compared to C males (*U* = 21, *p* = 0.01). C males played an average of 50.9 ± 5.1 times, PS males 34.3 ± 4.3 times. Females had no differences in frequency of play behavior (*U* = 65, *p* = 0.77). C females played on average 39.5 (35.7; 42.7) times, PS females 41.4 ± 3.8 times. It is interesting to note that C males had a greater frequency of play behavior than C females (*U* = 82, *p* = 0.02). This difference disappeared in the PS offspring (*U* = 47.5, *p* = 0.17). At the same time, the C offspring had no sex differences on the duration of play behavior (*p* = 0.29). The rat offspring had no differences in the latency of play behavior.

Differences in the duration and frequency of the play behavior were due to differences in such elements of social play as chasing, pinning, and boxing (Table 4). The offspring did not differ in pouncing.

**Table 4:** The elements of play behavior in rat offspring.

The elements of play behavior		Males			Females			Sex differences (*p* value)	
		C	PS	*p* value	C	PS	*p* value	C	PS
Total play behavior	Duration, s	134.2 ± 13.5	84.8 ± 10.7	**0.01**	104.7 ± 6.8	88.6 ± 9.8	0.70	0.29	0.99
	Frequency	50.9 ± 5.1	34.3 ± 4.3	**0.01**	39.5 ± 2.4	41.4 ± 3.8	0.77	**0.02**	0.17
	Latency, s	66.2 ± 7.3	71.5 ± 8.7	0.77	96.5 ± 22.4	94.9 ± 26.7	0.67	0.73	0.98
Pouncing	Duration, s	24.6 ± 5.0	23.4 ± 3.5	1	22.9 ± 5.5	18.0 ± 2.5	0.83	0.99	0.76
	Frequency	14.7 ± 2.4	14.5 ± 2.0	0.94	13.5 ± 2.6	12.9 ± 1.4	0.82	0.62	0.60
	Latency, s	79.8 ± 13.1	71.5 ± 12.4	0.87	127.3 ± 22.0	106.5 ± 33.4	0.63	0.12	0.35
Pinning	Duration, s	84.0 ± 10.3	42.1 ± 6.5	**0.004**	68.0 ± 9.2	51.7 ± 7.0	0.51	0.55	0.82
	Frequency	20.5 ± 2.9	11.3 ± 1.8	**0.03**	18.0 ± 1.9	17.0 ± 2.1	0.99	0.87	0.24
	Latency, s	96.1 ± 35.2	151.9 ± 18.1	0.05	140.1 ± 21.4	150.5 ± 32.4	1	0.28	0.44
Boxing	Duration, s	23.2 ± 3.4	12.1 ± 4.7	0.18	7.8 ± 3.7	16.2 ± 3.1	**0.01**	**0.005**	0.51
	Frequency	13.4 ± 1.6	5.5 ± 1.5	**0.01**	5.6 ± 1.0	9.0 ± 1.7	**0.02**	**0.004**	0.17
	Latency, s	87.2 ± 14.0	117.8 ± 39.8	0.31	131.4 ± 38.9	190.6 ± 36.5	0.62	0.47	0.66
Chasing	Duration, s	2.4 ± 0.5	0.74 ± 0.3	**0.01**	0.31 ± 0.29	0.48 ± 0.42	0.60	**0.01**	0.56
	Frequency	2.0 ± 0.5	0.55 ± 0.25	**0.004**	0.50 ± 0.26	0.50 ± 0.37	0.75	**0.01**	0.43
	Latency, s	339 ± 70	900 ± 68	**0.001**	850 ± 84	632 ± 104	0.46	**0.009**	0.27
Social exploration	Duration, s	40.9 ± 7.4	28.4 ± 5.6	0.28	32.0 ± 9.7	31.4 ± 4.6	0.38	0.25	0.98
	Frequency	12.9 ± 1.9	13.7 ± 2.3	0.99	11.6 ± 2.9	16.2 ± 1.7	0.47	0.98	0.84
	Latency, s	10.1 ± 2.1	10.2 ± 5.2	0.49	19.2 ± 4.1	15.6 ± 21.3	0.97	0.08	0.47

PS, prenatal stress offspring; C, control offspring. Statistically significant values (p < 0.05) are highlighted in bold.

Prenatal stress offspring had a decreased duration of the pinning (*F* = 12.5, *p* = 0.001) compared to C offspring at the expense of males (*p* = 0.004). C males spent an average of 84.0 ± 10.3 s for pinning, PS males 42.1 ± 6.5 s. C and PS females did not differ in the duration of the pinning (*p* = 0.51). The frequency of pinning had a difference (*F* = 5.34, *p* = 0.03) due to the difference in males (*p* = 0.03). C males had of 20.5 ± 2.9 times of pinning an average, PS males 11.3 ± 1.8 times. Females had no difference in frequency of pinning (*p* = 0.99). The rat offspring had no sex differences in pinning and no differences in latency of pinning.

We found differences in the duration of the boxing in females. PS females spent more time boxing [16.2 (9.4; 18.4) s], than C females [7.8(5.8; 8.6) s; *U* = 90, *p* = 0.01]. Males had no differences in the duration of the boxing [C males 23.2 ± 3.4 s, PS males 12.1(4.5; 28.1) s]. We also found sex differences in the duration of the boxing in C offspring. C males spent more time boxing than C females (*U* = 80, *p* = 0.005). PS offspring had no such difference. We also observed differences in the frequency of the boxing. PS males had a lower frequency of boxing [5.5(4.0; 13.0) times], than C males (13.4 ± 1.6 times; *U* = 22, *p* = 0.01), but PS females had a higher frequency of boxing [9.0(6.0; 12.0) times], than C females [5.6 ± 1.0 times; *U* = 96, *p* = 0.02]. We found sex differences in the frequency of the boxing only in C offspring – C males boxed more than C females (*U* = 88, *p* = 0.004), in PS offspring males and females did not differ. The latency of the boxing did not differ in the offspring.

Prenatal stress males had a shorter duration and frequency of the chasing [0.0(0.0; 1.4) s, 0.0(0.0; 1.0) times], than C males [2.4 ± 0.5 s, 2.0(1.0; 3.5) times; duration of the chasing *U* = 19, *p* = 0.01, frequency of the chasing *U* = 15, *p* = 0.004]. Females had no intergroup differences (duration of the chasing *U* = 68, *p* = 0.60, frequency of the chasing *U* = 65, *p* = 0.75). C females had chasing 0.3(0.0; 1.0) s, 0.5(0.0; 1.0) times, PS females 0.5(0.0; 2.1)s, 0.5(0.0;2.0)times. We also observed sex differences in C offspring – C males had greater duration (*U* = 84, *p* = 0.01) and frequency (*U* = 83, *p* = 0.01) of the chasing, than C females. The PS offspring had no such difference (duration of the chasing *U* = 57, *p* = 0.56, frequency of the chasing *U* = 54, *p* = 0.43). The latency of the chasing was strongly elevated in PS males [900(635; 900)с) compared with C males (339±71с; U=108, *p*=0.001). The C females (850(512; 900)с] и PS females [633(226; 900)с] did not differ on the latency of the chasing (*U* = 49, *p* = 0.46). We observed sex differences in latency of the chasing in C offspring. It was lower in C males than in C females (*U* = 15, *p* = 0.009). The PS offspring had no sex differences (*U* = 90, *p* = 0.27).

We found no differences in social exploration parameters.

### Effects of Neonatal Handling on Behavior

#### Effects of Neonatal Handling in Open-Field Test

We found in NH offspring the only statistically significant difference between PS and C offspring in the arena center crossing frequency. The hPS males crossed the center less frequently than the hC males (*U* = 150.5, *p* = 0.001). hPS males crossed the center on average 0 (0; 1) times, hC males crossed the center 2 (0; 3) times. We found no statistically significant differences in the other open-field test parameters. NH females demonstrated no differences in this test.

At the same time, NH factor influenced the parameters of behavior in the open-field test in both PS and С offspring.

hC offspring had a decrease in vertical activity compared to C offspring (males *U* = 157, *p* = 0.003; females *U* = 142, *p* = 0.002; Figure 2B). hC males had 3 (1; 5) rears on average, C males 7 (4; 11) rears, hC females 2 (0.25; 5) rears, and C females 10 (4; 13) rears. PS and hPS offspring were not statistically significant in rearing frequency.

It was demonstrated above that PS males had increased the total time of freezing. NH reduced the total time of freezing in hPS offspring to hC offspring values (Figure 2C). PS males had the total time of freezing on average 79.9 ± 13.1 s, hPS males 15.8 (5.3; 50.7) s (*U* = 81, *p* < 0.001). PS females had the total time of freezing on average 42.3 (31.2; 94.5) s, female hPS 24.8 (6.7; 38.0) s (*U* = 143, *p* = 0.02). hC and hPS offspring did not differ in the total time of freezing (males *U* = 330, *p* = 0.55; females *U* = 327, *p* = 0.85). Also, hC and C offspring did not differ significantly (males *U* = 259, *p* = 0.29; females *U* = 288, *p* = 0.83). However, PS and hPS offspring differed significantly in the total time of freezing (PS males *U* = 81, *p* < 0.001; PS females *U* = 143, *p* = 0.02). Therefore, NH restored the disturbed total time of freezing in the PS offspring to control values.

#### Effects of Neonatal Handling in Social Novelty Test

Neonatal handling had no effect on vertical activity in the social novelty test.

The LE was decreased in handled offspring compared with non-handled offspring on PNDs 22 and 33 (PND 22: *H* = 69.1, *p* < 0.001; PND 33: *H* = 49.7, *p* < 0.001). On PND 22, C offspring had the LE of 119.3 ± 14.1 s, hС offspring 20.9(10.0; 48.1)s, PS offspring 114.9 ± 12.9 s, and hPS offspring 17.3(10.4; 30.6). On PND 33, C offspring had the LE of 49.1(11.9; 80.6)s, hC offspring 18.5(9.1; 33.3)s, PS offspring 56.9 ± 7.9 s, and hPS offspring 8.0(4.9; 15.1). On PND 33, hPS males and hPS females exited their compartment more rapidly compared with hC males and hC females (hPS/hC males *U* = 168.5, *p* = 0.02; hPS/hC females *U* = 173, *p* = 0.004). Differences in the LE accounted for differences in the latency of contact with familiar and unfamiliar rats. The latency of contact with dams and unfamiliar female rats was reduced in all groups of handled offspring (the latency of contact with dams *H* = 62.9, *p* < 0.001; the latency of contact with unfamiliar female rats *H* = 35.1, *p* < 0.001). NH decreased the latency of contact with siblings only in hC males (*U* = 160, *p* = 0.04) and hPS males (*U* = 84.5, *p* = 0.009), compared to non-handled males. In addition, NH reduced the latency of contact with non-siblings in almost all offspring groups [C/hC males (*U* = 106, *p* = 0.001), C/hC females (*U* = 152, *p* = 0.09), PS/hPS males (*U* = 36, *p* < 0.001), and PS/hPS females (*U* = 33, *p* < 0.001)].

On PNDs 22 and 33, handled offspring had higher horizontal activity compared to non-handled offspring (PND 22: *F* = 90.1, *p* < 0.001; PND 33: *F* = 27.0, *p* < 0.001). On PND 22, the non-handled offspring had 25.2 ± 1.7 crossed squares; the handled offspring 46.6 ± 1.5 squares. On PND 33, non-handled offspring had 51.7 ± 2.6 crossed squares; handled offspring 66.4 ± 1.4 squares. The hPS offspring had greater horizontal activity compared with hC offspring on PND 22 (*F* = 9.8, *p* = 0.002). However, Tukey’s pairwise comparisons revealed differences only in females (*p* = 0.01; hC females 40.7 ± 2.2 squares, hPS females 53.8 ± 3.1 squares); hC and hPS males had no differences (*p* = 0.54).

We observed no sex differences in social interactions on PNDs 22 and 33.

Handled offspring had an increased total time of social contact compared to non-handled offspring on PND 22 (*U* = 6,164, *p* < 0.001) in almost all groups [C/hC males (*U* = 507, *p* < 0.001), C/hC females (*U* = 305, *p* = 0.05), PS/hPS males (*U* = 383, *p* < 0.001), and PS/hPS females (*U* = 347, *p* = 0.003); Figure 3A]. The NH had a significant effect on increasing the total time of social contact on PND 33 (*F* = 41.1, *p* < 0.001). However, Tukey’s corrections demonstrated that the total time of social contact increased only in hPS offspring compared to PS offspring [C/hC males (*p* = 0.39), C/hC females (*p* = 0.28), PS/hPS males (*p* < 0.001), and PS/hPS females (*p* = 0.001); Figure 3A].

The NH had a significant effect on the percentage of standing time in compartment with dams (*F* = 24.9, *p* < 0.001) and with unfamiliar female rats (*F* = 32.1, *p* < 0.001) on PND 22. The NH increased the percentage of standing time in compartment with dams in hC offspring compared to C offspring (*p* = 0.04) and in hPS offspring compared with PS offspring (*p* < 0.001); and at the same time, the NH restores this parameter in hPS offspring to hC offspring values (*p* = 0.56; Figure 3B). The NH reduced the percentage of standing time in compartment with unfamiliar female rats in hC offspring compared with C offspring (*p* = 0.02) and in hPS offspring compared with PS offspring (*p* < 0.001), and at the same time, NH restored this parameter in hPS offspring to hC offspring values (*p* = 0.65; Figure 3C). The NH had no effect on the percentage of standing time in compartment with siblings, but increased the percentage of standing time in compartment with non-siblings in hPS offspring to hC offspring values on PND 33 [PS/hPS males (*U* = 251, *p* = 0.02); PS/hPS females (*U* = 224, *p* = 0.03); Figure 3C].

The handled offspring had increased the standing time in the central compartment compared to the non-handled offspring on PND 22 (*U* = 2,503, *p* < 0.001; Figure 3D). However, such difference was confined to females. The handled females preferred to be in the central compartment more than non-handled females [PS/hPS females (*U* = 309, *p* = 0.02); C/hC females (*U* = 295, *p* = 0.02)]. Males had no differences. The NH had no effect on the standing time in the central compartment on PND 33.

## Discussion

The aim of our study was to determine the degree to which PS, induced by the negative emotional effect of variable frequency US, alters the development and behavior of rat offspring in infancy and juvenile age, as well as the maternal behavior of the stressed dams: (1) PS decreased vertical activity in dams, but did not alter maternal behavior scores, in the maternal behavior test. (2) PS has a negative effect on rat litter size. (3) The PS offspring had more weight than the control offspring since PND 6. The physical development indicators, such as fur appearance, pinna detachment, eye-opening, and lower incisors eruption did not differ between the groups. (4) PS did not affect the maturation of sensory-motor reflexes or motor behavior during the feeding period in rat pups. (5) PS decreased horizontal and vertical activity and increased the total time of freezing in the open-field test. The changes affected more males than females. (6) PS altered social preferences in pups during both the weaning and prepubertal periods. In PND 22, PS offspring preferred to spend more time in the compartment with unfamiliar females, and less with the dams, compared with control offspring. In PND 33, PS offspring preferred to spend less time in the compartment with non-sibling rats, compared to controls. (7) PS negatively affected social play behavior. PS males had decreased overall play behavior as well as components of play behavior (chasing, pinning, and boxing). PS females demonstrated no negative changes in this test. (8) NH improved PS offspring scores in the open-field test and social novelty test as a whole. NH reduced the total time of freezing in the open-field test in PS offspring (females and males) to control offspring values. NH decreased LE and increased horizontal activity on PND 22 in all experimental groups in the social novelty test. Also, NH increased the total time of social contact in almost all experimental groups on PND 22, in PS males and females on PND 33. NH restored social preference scores in the PS offspring to control values on PND 22 and 33.

### Maternal Behavior

We investigated the effects of PS on postpartum maternal behavior in order to test the hypothesis that offspring disorders may be induced by altered maternal behavior under the influence of stress and lack of maternal care. Indeed, most studies indicate that dams stressed during pregnancy exhibit less maternal care. For example, PS dams nursed their pups significantly less and spent less time with the pups compared to the control (Bosch et al., 2007), maternal contact and nursing behavior decreased during the light cycle (Bosch et al., 2007; Bourke et al., 2013). Also, stressed dams were characterized by decreased licking of pups (de Souza et al., 2012), increased frequency of being outside the nest (de Souza et al., 2012), and decreased arched-back nursing and nesting/grouping pups on PND 1–10 (Smith et al., 2004). However, Iturra-Mena et al. (2018) found no significant effect of stress during pregnancy on further maternal behavior. The same result was observed in our experiment: stress induced by a variable US did not affect maternal behavior. This suggests that impaired maternal behavior may not be responsible for the changes in the offspring. It is well known that the effects of PS on the offspring are largely due to the effects of maternal corticosterone overproduction, which is elevated during stress and crosses the placental barrier (Fowden and Forhead, 2015). Maternal corticosterone can negatively affect the fetus, including brain development and behavior (Fowden and Forhead, 2015; McGowan and Matthews, 2018). It is possible that the effect of stress induced by the variable US is due to this factor. This assumption requires additional research in the future.

It is worth noting that the first stage of the maternal behavioral test demonstrated that PS dams are characterized by reduced vertical activity (rearing frequency) in the arena. In the open-field test, reduced vertical activity can be interpreted as an indicator of anxious behavior (Sturman et al., 2018). However, despite possible anxious behavioral traits, the dams did not have impaired maternal behavior.

### Physical Development, Neurological Reflexes, and Motor Coordination

Variable frequency US-induced PS reduced rat litter size, but did not affect the fur appearance, pinna detachment, eye-opening, and lower incisors eruption in the offspring or the maturation of sensory-motor reflexes and motor coordination. However, many studies have demonstrated that PS can impair development in offspring. After chronic PS in the last week of pregnancy, delays in locomotor abilities, such as rotation on a flat surface, climbing an inclined screen, surface righting, and clinging to an inclined screen were observed (Meek et al., 2000). Prenatal bystander stress on 12–16 gestation days decreased positive geotaxis in females, but had no effect on male behavior (Mychasiuk et al., 2011). Chronic PS in the second half of gestation decreased anogenital distance and resulted in the earlier pinna detachment and eye-opening as well as faster righting. In a study by Nazeri et al. (2017), physical or psychological PS on gestation days 6–15 did not affect motor function, balance, and muscle strength in adolescent rat offspring (Nazeri et al., 2017). Also, PS had no significant effect on litter size or litter sex ratio (Secoli and Teixeira, 1998). In general, the results of studies on the effect of PS on offspring development at an early age are rather contradictory. It can be assumed that this is due to the fact that researchers use different types of stress and stressing protocols, it is also important that PS can be performed at different gestational ages. These factors may explain the differences in the results obtained. The effect of research protocols is demonstrated in the study of Fride and Weinstock (1984). After daily noise and light stress during the last week of gestation, PS rat offspring body weight did not differ from that of control rats, but postnatal development was significantly accelerated. At the same time, random stress induced general developmental delay in righting reflex, swimming behavior, and borderline significance for cliff avoidance. And daily stress throughout the gestation period resulted in decreased rat litter size and increased pup weight, but overall, their developmental rates did not differ from control (Fride and Weinstock, 1984). Similar results were obtained in our study using chronic stress throughout gestation. Patin et al. (2004) demonstrated that the effects of PS on the offspring depend largely on the stage of fetal development and, in particular, on the development stage of the central nervous system. In this study, pregnant female rats were exposed to acute or repeated stress (cat presence) on the 10th (when the neural tube was being formed) or 14th (when gross structures of the central nervous system began to differentiate) days of gestation. The average number of pups in a litter did not differ statistically between stressed and control rats. Acquisition of the pups’ precocious reflexes was delayed when the dams were subjected to the stressor at the 10th gestational day and, in most cases, was not when they were subjected to the stressor at the 14th gestational day. Repetitive stress had a greater effect than acute stress (Patin et al., 2004).

### Offspring Growth

Growth is the most important aspect of organism development that is related to the survival, maturation, and reproductive function of offspring (Berghänel et al., 2017). It is known that PS can have opposite effects on growth; growth can both be enhanced (Fride and Weinstock, 1984; Abe et al., 2007; Iturra-Mena et al., 2018), and attenuated (Drago et al., 1999; Mychasiuk et al., 2011; García-Vargas et al., 2019) under the PS influence. Therefore, there is no consensus on how PS affects offspring growth. A recent meta-analysis (Berghänel et al., 2017) proposed an integrative hypothesis: «developmental constraints and a counteracting adaptive growth plasticity work in opposition to drive prenatal maternal stress effects on growth». The developmental constraints hypothesis predicts that offspring exhibit reduced somatic growth in response to PS and this leads to later maturation, reduced body size, which in turn reduces lifetime reproductive success by reducing the reproductive life expectancy and reproductive rate. The adaptive growth plasticity hypothesis predicts faster growth and reproduction of offspring in response to PS. In this case, PS triggers a recalibration of offspring development toward a faster life strategy in which offspring grow faster, reach maturity earlier, reproduce faster, and have a shorter life span (Berghänel et al., 2017). In our study, PS offspring had a higher weight compared to control offspring starting at PND 6. One would assume that PS enhanced somatic growth of the offspring, however, it is worth taking into account the fact that PS females, on average, gave birth to fewer pups, and hence, the PS offspring received more resources from the mother than the control offspring, which could be the reason for the difference in weight at an early age. Additionally, body weight was not recorded in PND 1, in order to reduce the negative effect on the behavior of dams. This limitation does not allow accurate determination of the difference in birth weight and its association to litter size. This issue should be further investigated in future studies.

### Open-Field Test

Our experiment indicated that US PS decreased horizontal activity in the entire offspring, decreased vertical activity in males, and increased the total time of freezing in males in open-field test. We found no differences in the parameters of physical development and motor coordination, which demonstrates that no motor dysfunction was observed in PS offspring. Therefore, the observations of impaired motor activity made in the open-field test are most likely related to anxious behavior rather than to motor dysfunction. Increased freezing in PS males is also indicative of their increased anxiety. Interestingly, anxious behavior is more pronounced in PS males than in PS females. Many studies confirm that PS increases anxiety in animals both as juveniles and adults. According to Iturra-Mena et al. (2018), restraint PS had no effect on horizontal activity in male and female rats on PND 24, but significantly reduced the time that PS males spent in the center of the open-field. PS did not increase anxiety behavior in females in this study, which is consistent with our findings about sex differences (Iturra-Mena et al., 2018). Restraint PS increased anxiety in PS offspring rat on PND 22 in the open-field and elevated plus maze tests. Additionally, restraint PS altered the expression profile of anxiety-related genes. mGlu5 receptor expression increased and GABA_A_ receptor γ2 subunit expression decreased in the amygdala and this was consistent with increased anxious behavior in PS offspring (Laloux et al., 2012). Psychological PS induced by exposure to predator odor also increased anxious behavior in juvenile (PND 15) and adult (PND 76) (Green et al., 2018). Some works have indicated that PS induces anxious behavior not only in prepubertal age but also in adolescence (Badache et al., 2017; Nazeri et al., 2017; GhotbiRavandi et al., 2020) and adult (Zuena et al., 2008; Brunton, 2013; Green et al., 2018). The data on the effects of PS obtained in animals are consistent with studies in humans. Generalized anxiety and mood disorders are associated with stress, and their frequency increases in people exposed to PS (Weinstock, 2016).

### Social Novelty Test

One aspect of social behavior is the degree of interest in unfamiliar conspecifics, that is, the level of preference for social novelty. The desire to approach and explore unfamiliar conspecifics is inherent in social species, including rats, and it contributes to optimal social functioning. This is especially important for young individuals (Smith et al., 2015). Forms of social behavior change in rodents during postnatal development. The reorganization of sex hormone functioning as the organism matures is important in these changes. The interaction between mother and offspring is the most significant social interaction during the newborn and infancy (Veenema, 2012; Bell, 2018). These interactions are very intense and prolonged during offspring development (Veenema, 2012). The normal social environment of a developing rat pup includes not only the mother but also several siblings, with the mother and siblings providing different types of social experiences (Modlinska et al., 2018). Positive social interactions in postnatal development are an important part of the behavioral in rodents. Experiencing these behaviors influences normal development, mental health, establishment and maintenance of social structures, and reproductive success of the organism (Trezza et al., 2011).

Many studies have indicated that PS negatively affects the social activity of juvenile and adult offspring. For example, restraint stress of rat dams in the last week of gestation decreases the time of spontaneous social interactions in their pups on PND 24 (Iturra-Mena et al., 2018), and decreases the total time of social interactions in adult animals (Lee et al., 2007; de Souza et al., 2013). Social PS did not affect social preferences in adult offspring, but impaired social memory in females (Grundwald et al., 2016).

The study of the PS influence on rat social behavior is mostly investigated in adult animals. However, the pre-pubertal period is an important stage in the development and formation of social abilities. In the case of social behavior, the peri-weaning period (3rd and early 4th week after birth) is of primary importance. At this age, young rats continue to accept and establish contact not only with the mother but also with the siblings. Presumably, during this period, maternal influence on many aspects of life decreases, and contacts with siblings begin to be more important in the rat development (Modlinska et al., 2018). Notably, little research has focused on this period, so research on social behavior during this developmental period is needed (Modlinska et al., 2018).

The transition from infant to juvenile in rodents is usually defined by weaning on PND 21. However, pups tend to remain in close proximity to each other and the nest, gradually becoming more independent in their explorations (Bell, 2018). We performed a social novelty test involving dams and unfamiliar adult females on PND 22 when pups still have significant contact with dams. We found that at this stage of development, the PS offspring sought social novelty as they spent more time in the compartment with the unfamiliar females and less time in the compartment with the dams, compared to controls. On the one hand, one might assume, that PS, in this case, contributed to the social development of the rats, but some studies have demonstrated that high levels of novelty seeking can be maladaptive in both animals and humans because it predisposes them to potentially dangerous behaviors that can harm the individual (Smith et al., 2015). Repeated social novelty test on PND 33 with siblings and non-siblings confirmed the negative effect of PS on social novelty preference in rat pups. At this stage, PS offspring preferred to spend less time in the compartment with unfamiliar rat pups. The juvenile period in rats (28–40 PND) is characterized by increased involvement in peer-oriented social interactions, novelty seeking, and risk-taking behavior. Young rat males and females spend more time exploring an unfamiliar rat than a cage mate. The desire of young rats for novelty, including social novelty, is important during this period as rat’s transition from adolescence to adulthood and is in search of new territories, new food supplies, and potential partners (Smith et al., 2015). Therefore, the decreased interest in non-siblings in our experiment is evidence of the negative influence of PS on social functioning in rat pups. The increased interest of rat pups in an unfamiliar female on PND 22 is probably also not a normal behavior. As mentioned above, at this age, the rat pups continue to stay close to the nest and probably have a certain dependence on the mother. Contact with unfamiliar adult rats at this stage may have some danger for them, and therefore seeking contact with them may be undesirable.

### Social Play Behavior Test

Social play behavior is a complex behavior that requires both the ability to initiate social interactions and to respond appropriately to social signals (Cutuli et al., 2019). Social play involves the active interaction of two or more animals. This type of behavior is necessary for the development of social, cognitive, emotional, and motor abilities in both animals and humans (Vanderschuren and Trezza, 2014). Social play behavior is a highly satisfying activity; during play, neural mechanisms underlying positive emotions are engaged (Muhammad and Kolb, 2011; Vanderschuren and Trezza, 2014).

The structure of social play may differ between species or sexes (Vanderschuren and Trezza, 2014). The structure of social play behavior in rats has been described in several studies. It includes boxing/wrestling, pouncing, pinning, chasing, social grooming, and social sniffing (Vanderschuren et al., 1997). In rats, social play behavior usually begins with one rat “pouncing” on another rat, trying to grasp its neck. Two rats may “boxing,” that is, rapidly pushing, kicking, and grabbing at each other. If a rat flips another rat on its back, it is called “pinning.” During chasing, the rat chases the fleeing partner (Vanderschuren and Trezza, 2014; Cutuli et al., 2019). Social play begins to manifest on PND 17–19 in rats. It peaks between PND 28 and 40 and decreases as animals become sexually mature (Pellis and Pellis, 1990; Ward and Stehm, 1991; Vanderschuren and Trezza, 2014). We studied social play behavior in rats on PND 35 in our experiment.

Prenatal stress, caused by the action of variable frequency US, decreased duration, and frequency of the social play in males, but not in females. Differences in males were caused by variations in components of play, such as chasing, pinning, and boxing, but not pouncing. PS males exhibited less activity in these elements than control males. Controls had sex differences in the frequency of social play, with males playing more than females, which is consistent with findings from other studies (Ward and Stehm, 1991; Muhammad and Kolb, 2011; Argue and McCarthy, 2015; Zuena et al., 2016). Control males boxed and chased more frequently than control females. The PS offspring had no sex differences in duration and frequency of social play; these parameters in PS males decreased to the level of PS females. Therefore, PS eliminated sex differences in play behavior parameters and made male play behavior similar to the female type. Playing behavior in male rats was feminized. This phenomenon has been noted in other studies. Restraint PS combined with bright light in pregnant females (Ward and Stehm, 1991; Morley-Fletcher et al., 2003), the forced immobilization stress (Ohkawa, 1987), and placing pregnant females on an elevated platform (Muhammad and Kolb, 2011) reduced play behavior in male offspring. A study by Ward and Stehm (1991) noted a significant reduction in play behavior in PS males only in the “pouncing”; control females differed from control males only in this component (Ward and Stehm, 1991). Possible reasons for the above-described changes are described by Ward and Stehm (1991), who suggest that sex differences in play behavior may be related to plasma testosterone levels in males. Sexual dimorphism in social play is the result of higher testosterone levels in males than in females. The PS male fetus does not exhibit the spike in plasma testosterone levels observed in the unstressed male fetus on days 18 and 19 of gestation. Therefore, social play behavior in the prepubertal period may not be fully masculinized in the prenatal period (Ward and Stehm, 1991). There is evidence that PS can enhance play behavior in female offspring (Ohkawa, 1987), but this phenomenon was not observed in our experiment. There is a suggestion that PS may have an androgenic effect on the female fetus and an anti-androgenic effect on the male fetus. This pattern has been demonstrated in animal models and human (Barrett et al., 2014). Therefore, US PS had a negative effect on the social play behavior of the male offspring in our experiment. PS reduced the duration and frequency of social play in males on PND 35 to the level of females, indicating feminization of behavior in prepubertal PS males.

It should be taken into account that some studies have not demonstrated an effect of PS on the frequency or duration of play behavior (Takahashi et al., 1992; Himmler et al., 2014), so the question of the PS effect on social play behavior remains undetermined. It has previously been demonstrated that the frequency and structure of play behavior may differ at different ages in rats (Pellis and Pellis, 1990). Our study was performed with rats on PND 35. Future studies should investigate the effects of US PS on social play behavior in rats of different ages.

### Effects of Neonatal Handling on PS Offspring Behavior

The early postnatal period is a time of considerable brain plasticity, when even small exposures can lead to long-term programming of the immature brain, leading to normal or pathological processes in adulthood (Papaioannou et al., 2002; Raineki et al., 2014). Negative experiences during this period are associated with increased vulnerability to stress and poor physical and mental health, while positive experiences are conversely associated with resilience to stressful situations and good health (Raineki et al., 2014). Based on this data, an experimental paradigm known as “neonatal handling” was developed, in which rodent pups are briefly separated from their mothers for the first 3 weeks of life and exposed to a new environment (Levine et al., 1956; Papaioannou et al., 2002; Raineki et al., 2014). The NH effects include changes in hypothalamic-pituitary-adrenal axis function, improvement in the organism’s ability to cope with stress, and reducing anxious behavior (reduced emotionality; Papaioannou et al., 2002; Raineki et al., 2014; Siviy, 2018). At the same time, NH leads to ambiguous changes in learning and memory depending on the task, and negatively affects social behavior in early and adult life (Papaioannou et al., 2002; Raineki et al., 2014).

The classic NH procedure consists of placing pups separately from the dams in a new environment for 3 min and repeating this procedure daily with PND 1–20 (Raineki et al., 2014). The parameters of this model vary across studies in terms of the time of pup separation (1–15 min), number of exposure days and pups may also be exposed to additional stimuli during separation (e.g., grooming with a soft brush; Papaioannou et al., 2002; Garoflos et al., 2008; Bock et al., 2011; Zhang et al., 2012; Raineki et al., 2014; Akatsu et al., 2015; Siviy, 2018). In our experiment, the pups were subjected to various daily experimental manipulations during the first 3 weeks after birth, which included separating them from their mothers and transferring them to a new environment for testing. Such exposure may be comparable to the experimental NH procedure.

Currently, there are a sufficient number of studies on the NH effect on the behavior of healthy animals, but the number of studies on the NH effect on the PS offspring is limited. Existing works describe the NH effect on anxiety behavior and cognitive functions of the PS offspring, but there are no studies investigating social behavior under such conditions. In our experiment, we examined the NH effect on social and anxious behavior in PS rat offspring.

Neonatal handling decreased the total time of freezing in the PS offspring to the values of the control, indicating a decrease in anxious behavior. A decrease in LE under the NH influence in the social novelty test may also indicate a decrease in anxiety in rats. These results are consistent with studies by other authors in which NH had a positive effect in reducing anxiety in the PS rat offspring (Wakshlak and Weinstock, 1990; Bogoch et al., 2007; Raineki et al., 2014). At the same time, Akatsu et al. (2015) did not indicate a decrease in anxious behavior in PS mice under the NH influence (Akatsu et al., 2015).

We first demonstrated that NH can restore impaired social functions in PS offspring. NH positively affected social interactions in the PS offspring in the social novelty test. NH restored social preference scores in the PS offspring to control values and increased the total time of social contact in the PS offspring. Interestingly, NH increased the total time of social contact, increased standing time in the compartment with the dams, and decreased standing time in the compartment with unfamiliar females in the control rat on PND 22, which can probably be interpreted as a positive effect of NH. At the same time, existing studies for the most part suggest a negative effect of NH on social functions in healthy rodents (Raineki et al., 2014). For example, one study, NH impaired social memory in rats, decreased social exploratory interaction, and increased aggressive behavior in males (Todeschin et al., 2009). In another study, NH females presented deficits in maternal odor preference during infancy, but both males and females presented deficits in adult partner preference (Raineki et al., 2014).

Despite the described positive effects of NH, we found that NH increased in horizontal activity in the social novelty test on PND 22. Some studies have demonstrated that negative prenatal or postnatal exposure to various factors can lead to hyperactive behavior in animals. For example, preterm guinea pigs males had a higher distance traveled in the open-field arena compared to term males (Shaw et al., 2016). Effects of postweaning social isolation lead to hyperactivity in response to novelty in rats (Lapiz et al., 2003). This type of behavior is associated with clinical research on children suffering from attention-deficit/hyperactivity disorder (Shaw et al., 2016). This disease is associated with maternal stressful events during pregnancy (Ronald et al., 2011), poor maternal health, fetal post-maturity, long duration of labor, and maternal alcohol (Biederman and Faraone, 2005). Additionally, locomotor hyperactivity is one of the signs of impaired behavior when evaluating behavioral responses in models of schizophrenic-like behavior (Jones et al., 2011). Therefore, the presence of hyperactivity in rats under the action of NH in our experiment cannot be confidently described as a positive effect of NH. Unfortunately, the existing data on the effect of NH on hyperactive behavior are insufficient. However, there are studies that demonstrate a negative effect of NH on behavior. For example, HN is associated with rapid and increased palatable food ingestion, impaired behavioral flexibility, and fearless behavior to novel environments, a characteristic of impulsive behavior that is a key component of many psychiatric disorders (Lazzaretti et al., 2016).

Our experiment indicated that NH had a positive effect on anxiety and social behavior in PS offspring, which were altered in them. However, NH induced a hyperactive behavioral phenotype in the offspring, which is associated to a greater extent with pathological behavioral responses.

### Conclusion

In conclusion, the results presented here provide further evidence of the deleterious effects of prenatal exposure to maternal psychological stress on offspring. PS induced by exposure to variable frequency ultrasound throughout gestation influenced the development of rat pups during infancy. PS offspring had greater body weight compared to control offspring. Under the PS influence, anxiety was increased in rat offspring, especially in males. PS negatively affected the social preferences in male and female pups and impaired the social play behavior in males. Neonatal handling reduced anxiety and restored social functions in PS offspring, but induced hyperactivity in rat offspring. Our study demonstrated that the action of variable ultrasound during pregnancy induces changes in the offspring similar to those in traditional ways of stressing pregnant females. This method can be used in the study of the PS action.

## Data Availability Statement

The raw data supporting the conclusions of this article will be made available by the authors, without undue reservation.

## Ethics Statement

The animal study was reviewed and approved by Housing conditions, and all experimental procedures were set up and maintained in accordance with Directive 2010/63/EU of 22 September 2010 and approved by the local ethical committee of V.P. Serbsky National Medical Research Center for Psychiatry and Narcology.

## Author Contributions

OA, VU, YZ, and EZ planned and carried out the experiments, and wrote the manuscript. AM, ZS, and VC revised the manuscript. All authors contributed to the article and approved the submitted version.

### Conflict of interest

The authors declare that the research was conducted in the absence of any commercial or financial relationships that could be construed as a potential conflict of interest.

## Abbreviations

NH: Neonatal handling
PND: Postnatal day
PS: Prenatal stress
US: Ultrasound
